# Severity Levels of Disability Among Older Adults in Low- and Middle-Income Countries: Results From the Study on Global Ageing and Adult Health (SAGE)

**DOI:** 10.3389/fmed.2020.562963

**Published:** 2020-10-15

**Authors:** Aarón Salinas-Rodríguez, Ana Rivera-Almaraz, Ashley Scott, Betty Manrique-Espinoza

**Affiliations:** ^1^Center for Evaluation Research and Surveys, National Institute of Public Health, Cuernavaca, Mexico; ^2^Division of Chronic Disease Prevention and Control, Boston Public Health Commission, Boston, MA, United States

**Keywords:** disability, latent class analysis, geriatric syndromes, low- and middle-income countries, older adults

## Abstract

**Background:** Recent studies suggest the importance of distinguishing the severity levels of disability in the older adult population. However, there is still no consensus regarding an optimal classification. Few studies have estimated the prevalence of severe disability, and the results have been confined to high-income countries. There is no evidence for low- and middle-income countries (LMICs). Therefore, the aim of this study was to provide estimates of the levels of severity associated with disability in older adult populations in LMICs and to examine their relationship with health and socioeconomic factors.

**Methods:** We used data from the Study on global AGEing and adult health (SAGE), wave 1 (2007–2010). Nationally representative samples of adults over 50 years from China, Ghana, India, Mexico, Russian Federation, and South Africa were analyzed (*n* = 33,641). We measured disability using the World Health Organization Disability Assessment Instrument version 2.0 (WHODAS 2.0). Disability levels according to severity were identified through the use of latent class analysis. Socioeconomic and health factors associated with severe disability were estimated using ordinal logistic regression models.

**Results:** We identified four groups of older adult: (1) without disability, 43.4%; (2) mild disability, 33.3%; (3) moderate disability, 15.3%; and (4) severe disability, 8.0%. These results were heterogeneous for the six countries analyzed. Education and socioeconomic status were significantly associated with severe disability along with the following chronic conditions: angina, arthritis, asthma, cataracts, chronic obstructive pulmonary disease, depression, diabetes, and stroke. Severe disability was also associated with the frailty status, sarcopenia, and mild cognitive impairment.

**Conclusions:** In this study, we estimated severity levels of disability for the older adult population in LMICs. Our results show that severe disability affects 8% of older adult, and that there are important socioeconomic and health factors associated with this condition. Measuring the severity of disability is a critical element to study the causes and consequences of aging. Moreover, the identification of older adult with severe disability is vital to design prevention programs, modify interventions, or develop enabling environments.

## Introduction

Functional impairment and disability are important health-related conditions in the older adult population (1, 2). Burden imposed by disability in old age is one of the major challenges faced by healthcare systems worldwide not only in high-income countries (HICs) but also in low- and middle-income countries (LMICs) (3). Disability can occur simultaneously with some geriatric syndromes (falls, frailty, cognitive impairment) (4, 5) and chronic conditions (hypertension, myocardial infarction, arthritis, diabetes, stroke) (6, 7) and is a major risk factor for adverse outcomes such as falls (8), hospitalizations (9), and mortality (10).

Disability has many conceptual and empirical definitions (11, 12). One of the most used is from the World Health Organization (WHO) based on the International Classification of Functioning, Disability, and Health (ICF) (13) and operationalized in the WHO Disability Assessment Instrument (WHODAS) instrument. WHODAS has been well-validated as an instrument for measuring disability across multiple countries (14, 15). However, no well-established cutoffs exist for binary or ordinal classifications.

Several studies have estimated the prevalence of disability using the recommended 90th percentile as a threshold for modeling WHODAS 2.0 scores as a dichotomized outcome (16–18). This binary classification approach could be useful, for example, to estimate disability prevalence and target specific interventions among the disabled. Nonetheless, such an approach does not consider the heterogeneity associated with disability severity levels frequently observed in the older adult population.

Regarding ordinal classifications, some studies have utilized the ICF categories (13) to estimate the severity levels of disability (17, 19, 20). However, such approach can only be used when there are calibrated assessment instruments or other standards (13, p. 230). To further emphasize, this evidence is concentrated in HICs, and scarce research has been conducted in LMICs, especially among older adults. Aside from the ICF-recommended categories, other efforts have been made to create ordinal classifications based on statistical methodologies such as latent class analysis (LCA), but again evidence comes from HICs (21, 22).

Studies in LMICs have already analyzed disability in older adults. Most of them use scales that focus on the physical component of disability, particularly the limitations for activities (basic and instrumental) of daily life (23–25). Other studies have used the WHODAS 2.0 to assess disability. In these studies, two analytical strategies have prevailed. First, the disability is modeled in a continuum range (capture by the WHODAS score), in which higher scores represent a higher level of disability (26, 27). Second, a cutoff is applied to the WHODAS score (using the 90th percentile) to generate a dichotomous variable indicating disability (18, 28). Disability prevalence, using limitations in daily living activities, has ranged from 16 to 54% (25), while with the WHODAS 2.0 data (using the 90th percentile), prevalence ranges from 25 to 55% (28). Notably, none of these studies estimated the severity levels associated with disability.

Therefore, the aims of this study were to provide estimates of the levels of severity associated with disability in older adult populations in LMICs and to assess disability's association with health and socioeconomic factors. Our hypothesis is that the heterogeneity of disability levels could be described in distinct patterns based on the observed responses to the 12 categorical items of WHODAS 2.0. Specifically, we used LCA) to identify homogeneous classes, or groups, related to the severity of disability. To our knowledge, this study is the first attempt to analyze the severity levels of disability among older adults in a group of LMICs.

## Materials and Methods

### Sample

Cross-sectional data from the WHO longitudinal multi-country Study on global AGEing and adult health (SAGE) Wave 1, conducted in China, Ghana, India, Mexico, Russian Federation, and South Africa during 2007–2010, was used for the analyses. At the time of data collection for Wave 1, SAGE included a mix of one low-income (Ghana), two lower middle-income (China and India), and three upper middle-income countries (South Africa, Mexico, and Russian Federation). These countries vary in stages for demographic and epidemiological transitions but are experiencing a rapid rise in their older adult populations (29).

SAGE employed a multistage cluster design with samples drawn from an updated national sampling frame. Face-to-face interviews were used to capture respondent information. A detailed description of the study, sample design, and weighting is provided elsewhere (29, 30). SAGE was designed as a study representative of the population aged 50 years or older in each participating country. The wave 1 six-country pooled sample consisted of 36,428 respondents aged 50+ years. The analytical sample for this study included 33,641 respondents with complete data (92.3%). Sample sizes for each country were: China, *n* = 12,969; Ghana, *n* = 4,302; India, *n* = 6,559; Mexico, *n* = 2,211; Russia, *n* = 3,919; and South Africa, *n* = 3,681.

### Outcome Variable

We measured disability using the cross-culturally validated 12-item version of the WHODAS Version 2.0 (WHODAS 2.0). The WHODAS 2.0 scale is widely used to measure last-month limitations in activity and daily-life participation. It covers six domains explored through a total of 12 items (two per domain): [1] cognition and communication, [2] self-care, [3] mobility, [4] interpersonal relations, [5] life activities, and [6] participation. The results of the 12 items are added up to obtain a global score expressed on a continuous scale from 0 (no disability) to 100 (full disability) (31).

### Covariates

Covariates were categorized as follows: sex (1 = female), age group (50–59, 60–69, 70–79, ≥80), marital status (1 = currently married/cohabiting or other), years of education (no formal schooling, 1–5 years, 6–9 years, and 10 or more years of schooling). Socioeconomic status (SES) of the household was derived using the WHO standard approach to estimate permanent income from household ownership of durable goods, dwelling characteristics (type of floors, walls, and cooking stove), and access to services such as water, sanitation, and electricity (32, 33). SES was transformed into quintiles (country specific), with the lowest quintile (Q1) indicating the poorest households and the highest quintile (Q5) indicating the richest households. We also include the place of residence (rural vs. urban) and a dummy variable for the countries, using China as the reference category.

We used the list of nine chronic diseases included in the SAGE study. The three following conditions were measured according to self-reported medical diagnoses: diabetes, stroke, and cataracts. Another five conditions were estimated through algorithms for symptomatology and self-reported treatment: angina, arthritis, chronic obstructive pulmonary disease, asthma, and depression. Finally, hypertension was determined by either blood pressure measurement and/or self-reported treatment. A detailed description of definition and operationalization of these diseases has been published elsewhere (34).

We included three variables related to geriatric syndromes. Frailty status was determined using a modified frailty phenotype based on the criteria proposed by Fried et al. (35) that covers five components: weight loss, exhaustion, low physical activity, slow walking speed, and weakness. Respondents were considered frail if they met three or more of these criteria, prefrail if they met one or two, and not frail or robust if they met none of the above criteria. Since the original cutoff points of the frailty phenotype have not been validated in LMICs, we used the lowest quintile approach (country-specific) for the items measured on a continuous scale (36). Details of the application on this measurement of frailty in the SAGE sample have been published elsewhere (37). We briefly describe its measurement here. Gait speed was measured by recording the time taken in seconds to walk 4 m at a normal speed. Slow gait speed was defined by the lowest quintile, stratified by sex and height. The presence of the weight loss criterion was based on the lowest quintile of body mass index (BMI). Grip strength was assessed with a handheld dynamometer using the sum of the highest values of two measurements on each hand. The lowest quintile stratified by sex and BMI was applied as a cutoff to indicate low grip strength. Exhaustion was measured on a 5-point Likert scale by asking respondents whether they had enough energy for daily activities. This criterion was considered present if participants answered, “Not at all” or “A little.” Finally, physical activity was assessed using the WHO Global Physical Activity Questionnaire (GPAQ). The low physical activity criterion was present if activity <600 metabolic equivalent of task (MET) minutes a week as defined by the GPAQ.

We also used sarcopenia as an additional covariate. According to previous publications, we defined the presence of sarcopenia as having low skeletal muscle mass (SMM) as reflected by lower skeletal muscle mass index (SMI) and either a slow gait speed or weak handgrip strength. Details of this variable have been published using the SAGE study sample as well as the description of the specific algorithms used to define sarcopenia status in the older adult population (38). Briefly, this variable was measured as follows. Slow gait speed and low handgrip strength were defined according to the procedure described in the previous paragraph. Regarding SMM, calculations were made as appendicular skeletal muscle mass (ASM) using the equation proposed by Lee et al. (39). From this, the SMI was obtained, dividing the ASM by the BMI (40). We defined the low SMM based on the presence of low SMI, which was established by the lowest quintile (country-specific) of the SMI based on sex-stratified values.

Finally, mild cognitive impairment (MCI) was also included. Based on recommendations from the National Institute of Aging-Alzheimer's Association in previous work with the SAGE database, an algorithm was used to generate the MCI variable (41). In sum, older adults who fulfilled the following criteria were considered to have MCI: (a) concern about a change in cognition; (b) objective evidence of impairment in one or more of the next cognitive domains: learning and episodic memory, attention and working memory, and verbal fluency; and (c) dependence in functional abilities.

### Statistical Analysis

We used pooled data from all six countries to conduct the data analysis and summarized sample descriptive characteristics by country. Given that our aims were to provide severity level estimates associated with disability and to examine their associated factors, we conducted the analysis in the two phases described below.

### Identification of Severity Levels of Disability

We used LCA to identify potential groups of older adults according to severity levels of disability. LCA is similar to cluster analysis in that both techniques aim to identify groups of individuals who have a similar response pattern to a set of observed variables. The main difference between these two methodologies is that LCA is a modeling-based approach (42).

The aim of LCA is to estimate the number of classes of an underlying categorical latent variable that accounts for the association patterns between categorical observed variables. In our case, LCA creates subgroups of older adults who respond in a similar way to the observed variables of WHODAS 2.0. In this model, there are two parameters of interest: (a) class membership probabilities and (b) the probability of response to each observed variable given the membership of the latent class. For LCA models, the posterior probabilities indicate the probability that an individual will be assigned a given class, and individuals are classified into their highest-class probability.

We determined the number of classes based on three criteria (43). First, we used the adjusted Bayesian Information Criteria (aBIC), where lower values on this fit statistic indicate a better model fit, the Entropy value, which indicates the precision of classification, and the bootstrap likelihood ratio test (BLRT), which compares a *k* class solution to *k-1* class solution where *k* is a given number of latent classes (42). Second, using the WHODAS continuous score as an auxiliary variable, we graphically assessed the LCA solution for k classes to verify whether solutions highlight informative and exhaustive differences between classes. Lastly, we used the principles of interpretability, parsimony, and similarity of latent classes size.

### Factors Associated With Severity Levels of Disability

Given the ordinal nature of the categories associated with our dependent variable (the number of classes identified in LCA), we used a multivariate ordinal logistic regression model to identify the correlates of severity levels of disability.

Statistical analyses accounting for the complex survey design, including verification of the proportional odds assumption, were carried out using Stata 16.0 software (StataCorp LP, Texas, USA). Differences were considered statistically significant if *p* < 0.05 and 95% confidence intervals (CIs) were given.

## Results

### Sample Characteristics

The sociodemographic and health characteristics (weighted percentages using the complex sampling design) of respondents are presented in Table 1. A total of 33,641 older adults age 50 and over were included in the subsequent analysis.

**Table 1:** Sociodemographic and health characteristics of older adults by country and pooled data^a^.

	**Country**						**Pooled data**
	**China (*n* = 12,969)**	**Ghana (*n* = 4,302)**	**India (*n* = 6,559)**	**Mexico (*n* = 2,211)**	**Russia (*n* = 3,919)**	**South Africa (*n* = 3,681)**	**All countries (*n* = 33,641)**
**Covariates**							
Sex, female	50.2	47.6	49.0	53.4	61.0	56.1	52.2
**Age group, years**							
50–59	45.2	39.8	48.6	49.1	45.2	49.8	46.7
60–69	31.9	27.5	30.9	25.8	24.6	30.6	29.7
70–79	18.6	23.1	16.0	17.8	21.8	14.0	18.1
≥80	4.3	9.6	4.5	7.3	8.4	5.6	5.5
Marital status, with couple	85.4	59.3	76.9	73.0	58.3	55.8	75.4
**Years of education**							
No formal education	22.5	54.2	51.4	17.2	0.7	24.8	28.6
1–5	25.5	8.7	19.2	38.8	6.1	21.3	20.4
6–9	35.3	8.3	13.1	33.7	20.5	30.3	24.4
≥10	16.7	28.8	16.3	10.3	72.7	23.6	26.6
**Income quintile**							
Q1	16.2	18.3	21.9	14.4	19.1	21.2	18.7
Q2	18.4	19.4	21.5	24.3	18.9	20.1	20.1
Q3	20.2	20.6	19.7	17.7	18.5	19.0	19.5
Q4	22.9	20.4	17.9	15.5	23.0	19.5	20.5
Q5	22.3	21.3	19.0	28.1	20.5	20.2	21.2
Residence, rural	52.4	59.0	71.1	21.5	27.2	35.1	51.5
**Chronic diseases**							
Angina	7.3	10.4	13.7	3.6	33.7	7.9	14.0
Arthritis	20.1	23.2	23.5	12.5	33.4	26.8	23.4
Asthma	3.9	3.7	11.0	3.9	6.2	7.0	6.8
Cataract	8.0	5.3	17.5	10.0	12.5	4.4	12.1
Chronic obstructive pulmonary disease (COPD)	9.0	3.5	15.9	10.7	19.1	7.1	13.2
Depression	1.5	8.3	15.2	10.8	4.8	3.7	7.6
Diabetes	6.6	3.8	6.9	17.6	7.0	9.3	7.6
Hypertension	61.4	60.7	34.6	58.5	69.3	78.5	54.0
Stroke	3.1	2.8	2.0	4.3	4.8	4.0	3.1
**Geriatric syndromes**							
**Frailty**							
Non-frail	40.5	32.8	36.6	43.3	31.5	22.7	37.0
Pre frail	51.8	55.6	50.5	48.9	56.2	59.9	52.3
Frail	7.7	11.6	13.0	7.8	12.2	17.4	10.7
Sarcopenia	10.6	14.1	20.1	14.5	14.2	16.2	15.0
Mild cognitive impairment	27.0	7.9	14.0	16.5	10.9	7.0	18.0
Multimorbidity (two or more diseases)	30.6	31.8	38.7	35.1	55.6	37.5	38.4

a *Cells are percentages*.

In general, most of the older adults were women, belonged to the youngest age group (50–59 years) and had a partner or were married at the time of the survey. In terms of chronic conditions, the most prevalent were hypertension (54%), arthritis (23.4%), and angina (14%). As for geriatric syndromes, frailty was observed in 10.7% of the sample, sarcopenia in 15%, mild cognitive impairment in 18%, and multimorbidity in 38.4%.

We also observed some heterogeneity for the six countries analyzed. The highest prevalence of chronic diseases was observed in Russia, and those of frailty and sarcopenia in India and South Africa. Meanwhile, the percentage of rural population was highest in India, Ghana, and China, and the lowest was in Russia and Mexico. Finally, the highest level of schooling was observed in Russia and the lowest was in Mexico.

### Application of Latent Class Analysis

We adjusted models from one to six classes selecting the four-class model based on indices of fit as well as a graphical inspection of the WHODAS 2.0 score along the classes observed. Supplementary Table 1 shows the results of the fit indices. Based on BLRT, the best model was with four classes, although for the Entropy, the best was the five-class model. We selected the four-class model, in spite of BLRT, because the four-class solution exhibited clearer separation between latent classes according to the distribution of WHODAS 2.0 score than the five-class solution (Supplementary Figure 1). We also present in Supplementary Table 2 and Supplementary Figure 2 the response proportions for each item of WHODAS 2.0 and the probabilities of severe and extreme difficulty for activity in each of its six domains, respectively.

Supplementary Figure 3 shows our semantic interpretation of the four classes identified. For the class with scores of WHODAS 2.0 closest to zero (mean = 3.5, 95% CI: 3.2–3.8), we labeled it as “no disability.” Following a similar reasoning and using the WHODAS 2.0 score as auxiliary variable, the second class (mean = 18.9, 95% CI: 18.3–19.4) was interpreted as “mild disability,” third as “moderate disability” (mean = 37.9, 95% CI: 37.4–38.4), and fourth as “severe disability” (mean = 59.7, 95% CI: 58.6–60.8).

### Severity Levels of Disability

**Figure 1:**
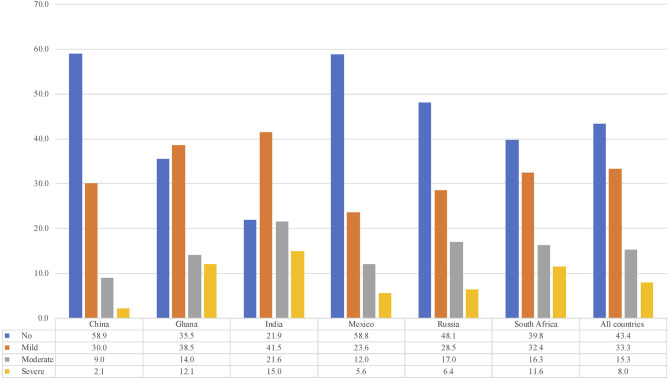
Prevalence of disability levels by country.

The results of the estimation of disability severity levels are shown in Figure 1. For the pooled data, prevalence rates of each severity level were: no disability, 43.4%; mild disability, 33.3%; moderate disability, 15.3%; and severe disability, 8.0%.

However, we also observed significant differences between countries regarding the prevalence of severe disability. China had the lowest prevalence (2.1%), followed by Mexico (5.6%) and Russia (6.4%). Meanwhile, India (15.0%), Ghana (12.1%), and South Africa (11.6%) showed the highest prevalence (F = 52.9, *p* < 0.01). Similar heterogeneity patterns were observed for the rest of the severity levels.

### Association of Severity Levels of Disability With Health and Socioeconomic Factors

Table 2 shows the results of the ordinal logistic regression model. The following sociodemographic variables were associated with a higher probability of having a greater severity of disability: being woman [OR (odds ratio) = 1.44], older than 50–59 years (OR = 1.42, age group 60–69; OR = 2.32, age group 70–79; OR = 5.06, age group 80+), and living in rural areas (OR = 1.32). On the contrary, higher levels of schooling (OR = 0.92, 1–5 schooling years group; OR = 0.71, 6–9 schooling years group; OR = 0.49, 10+ schooling years group), and SES (OR = 0.83, Q2; OR = 0.80, Q3; OR = 0.69, Q4; OR = 0.55, Q5) were associated with lower probabilities.

**Table 2:** Results of the ordinal logistic regression model for disability severity levels.

**Covariate**	**OR**	**95% CI**	
Sex (female = 1)	1.44	1.27,	1.62
**Age (reference: 50–59 years)**			
60–69	1.42	1.26,	1.61
70–79	2.32	2.01,	2.67
≥80	5.06	4.05,	6.32
Marital status (with couple = 1)	0.94	0.81,	1.09
**Years of formal education (reference: no formal education)**			
1–5	0.92	0.80,	1.05
6–9	0.71	0.61,	0.83
≥10	0.49	0.41,	0.59
**SES (reference: Q1)**			
Q2	0.83	0.69,	1.00
Q3	0.80	0.67,	0.97
Q4	0.69	0.57,	0.83
Q5	0.55	0.45,	0.67
Residence (rural = 1)	1.32	1.08,	1.61
**Geriatric syndromes**			
**Frailty (reference: non-frail)**			
Pre frail	1.66	1.47,	1.88
Frail	4.92	3.79,	6.39
Sarcopenia	1.25	1.09,	1.44
Mild cognitive impairment	1.85	1.62	2.11
**Chronic diseases**			
Angina	1.58	1.34,	1.87
Arthritis	1.72	1.52,	1.95
Asthma	1.48	1.24,	1.75
Cataract	1.26	1.11,	1.43
Chronic obstructive pulmonary disease (COPD)	1.89	1.65,	2.16
Depression	2.00	1.64,	2.44
Diabetes	1.23	1.05,	1.45
Hypertension	1.11	1.00,	1.23
Stroke	2.91	2.24,	3.79
**Country (reference: China)**			
India	4.55	3.78,	5.48
Mexico	1.16	0.75,	1.81
Russia	1.68	1.24,	2.28
South Africa	2.76	2.27,	3.36
Ghana	2.73	2.24,	3.32
Intercept 1	1.23	0.95,	1.52
Intercept 2	3.34	3.05,	3.64
Intercept 3	5.05	4.75,	5.35

For the geriatric syndromes, being pre-frail or frail (OR = 1.66 for pre-frail older adults; OR = 4.92 for frail), sarcopenia (OR = 1.25), and cognitive impairment (OR = 1.85) were associated with higher severity levels of disability. Regarding chronic conditions, angina (OR = 1.58), arthritis (OR = 1.72), asthma (OR = 1.48), cataract (OR = 1.26), lung disease (OR = 1.89), depression (OR = 2.00), diabetes (OR = 1.23), and stroke (OR = 2.91) were also significantly associated (Table 2).

Finally, and using China as the reference category, we observed significant differences between countries related to severity of disability, except for Mexico (OR = 1.16, 95% CI: 0.75–1.81).

## Discussion

### Main Findings

In this study, we found four groups of older adults according to the severity levels of disability. Almost half of the respondents were classified as no disability and one third with mild disabilities. Meanwhile, moderate or severe disability levels affected a quarter of the elderly population in the LMICs analyzed.

However, we also observed significant heterogeneity among the countries. The prevalence of severe disability was lower in China, Mexico, and Russia and between three to five times higher for South Africa, Ghana, and India. Notably, almost 60% of older adults from China and Mexico were disability-free.

Higher levels of schooling and SES were associated with lower severity levels of disability. Frailty was the condition that resulted in a stronger association with severe disability, and chronic conditions strongly associated with disability were stroke, depression, and chronic obstructive pulmonary disease.

### Comparison With Findings From Previous Studies

Some studies have estimated the severity levels of disability using WHODAS 2.0 (either 12- or 36-item version), following two general approaches. First, using the ICF-recommended classification, a population is classified as: no disability [0–4], mild disability [5–24], moderate disability [25–49], severe disability [50–95], and extreme disability [96–100]. Second, using some multivariate statistical method, individuals are classified according to the association pattern observed among the items of WHODAS 2.0.

Regarding the first approach, one study with older adults aged 60–70 in Poland found a prevalence of severe or extreme disability of 6.3% (44). Two additional studies, conducted in Spain, one with older adults aged ≥75 reported a prevalence of 10.14%, and the other with individuals aged ≥50 found a prevalence of 8% (19, 45). These results are similar to our estimation of severe disability, particularly that of Almazán-Isla et al. (45), whose study population has the same age range as that of our older adult sample.

For the second approach, we found three studies that used WHODAS 2.0 and applied LCA to identify groups of individuals with different profiles of disability. A first study conducted in Italy with adults aged ≥65 identified four groups of individuals: without disability (60.8%), with difficulties in movements (21.2%), with difficulties in movements and daily tasks (11.4%), and with very low functioning level (6.6%), this last group being equivalent to our estimation to severe disability (46). A second study with older English adults aged 50 years found a similar LCA solution to ours, with four groups of individuals identified: no disability, mild disability, moderate disability, and severe disability (22). However, there are two substantial differences that must be noted. First, this was a longitudinal study (no prevalence of these classes was provided); and second, latent classes were estimated using a set of 50 binary and categorical variables identified as indicators of disability, not only WHODAS 2.0. Even so, it is worth noting that by applying the same statistical technique (LCA), they found the same solution with four classes interpreted in the same sense as we interpret our results. Finally, a third study conducted in Canada, although with adults aged 18 or more, also found a four-class solution that was interpreted as: (1) pervasive disability (19.1%); (2) physical disability (10.8%); (3) emotional, cognitive, or interpersonal disability (28.6%); and (4) no/low disability (41.5%) (21). The class of pervasive disability could be somewhat similar to our severe disability group; however, its prevalence was quite high. This could be explained because the sample for this study was constructed from four distinct studies and included people with emotional or behavioral issues (excessive drinking, illegal substance use, or conflictive intimate partner relationships).

Regarding evidence in LMICs, several studies have looked at disability among older adults. However, it is difficult to benchmark our results because no other study had a similar scope. Even so, we chose four studies that are different but of relevance for triangulation. Stewart Williams et al. (27) analyzed, as a secondary aim, the factors associated with disability, emphasizing falls as main exposure, using the data of the SAGE study. They reported that the WHODAS score was higher in subjects who reported fall-related injury in the past year (5.62 points higher). However, they did not analyze the presence or the severity levels of disability, but rather the continuous WHODAS score. In that sense, our results are different because we classified the population according to severe disability levels and identified the most vulnerable group of older adults, i.e., those with a severe disability. In another study that also uses the SAGE study data, Biritwum et al. (28) estimate the frailty and disability prevalence as well as their association with socioeconomic factors. Disability was measured using the WHODAS questionnaire, and the presence of disability was defined, taking the 90th percentile of WHODAS score as the cutoff. The prevalence of disability varies according to the countries analyzed, with the lowest being 25% for China and the highest for India with 55%. They also reported that sex, age, education, and wealth were significantly associated with the presence of disability. These prevalences may include all individuals with some degree of disability (mild, moderate, and severe), and hence their numbers look very high. However, and given that different analytical methodologies were used, the comparison with our results is not adequate. Even so, the results are consistent as China reports the lowest prevalence for any level of disability severity, and India reports the highest. Regarding associated factors, our study also found significant associations with sex, age, education, and wealth. Nevertheless, our results go beyond these factors, including health conditions (expressed by nine chronic conditions) and three geriatric syndromes (frailty, sarcopenia, and cognitive impairment), with all of them significantly associated with the severity levels of disability.

One more study that also uses the SAGE study's data estimated the prevalence and associated factors of disability but used limitations in basic activities of daily living (25). The results are consistent with ours and those of Biritwum et al. (28), where China shows the lowest prevalence of disability (16%) and India shows the highest (55%). Overall, older age, multimorbidity, and depression were associated with disability; all these factors with significant associations were also observed in our study. It is important to note that in this study, only the physical component of disability was explored, while in our study, different dimensions of disability are incorporated. Finally, Sousa et al. (18) estimated the contribution of chronic diseases to disability using data from the 10/66 Dementia Research Group study. Disability was measured using the WHODAS 2.0 questionnaire, and the presence of disability was determined using the 90th percentile of the WHODAS score. The prevalence of disability was not reported, but the mean WHODAS score was. Of seven included countries, China, India, and Mexico coincide with those of the SAGE study. China had the lowest levels of disability (mean WHODAS score = 8, SD = 14.5), and India had the highest (mean WHODAS score = 28, SD = 18.3). Additionally, the chronic conditions that had the greatest contribution to disability were dementia, stroke, limb deterioration, arthritis, and depression. Although the analytical approach was different, the findings coincide in the effect that certain chronic diseases have on the presence of disability [in the study by (18)] or their severity levels according to our study. It should be noted that severity levels of disability were not estimated, and the geriatric syndromes such as frailty and sarcopenia were not included in either study.

As for variables associated with severe disability, we found that two indicators of better SES (schooling and permanent income) were protective factors for the presence of higher levels of severe disability. In that sense, it is believed that SES and disability reinforce each other, operating in a pernicious cycle (47). A recent systematic review, with studies in LMICs, found a strong evidence for a link between disability and poverty (48).

Consistent with previous studies, three geriatric syndromes (frailty, sarcopenia, and cognitive impairment) were associated with severe levels of disability (49). Although the mechanisms through which geriatric syndromes contribute to disablement are not clear, it is possible that these syndromes are indicators of some underlying pathology, a deterioration of the autoregulatory system or the presence of other risk factors (50).

The associations between the nine chronic conditions included in this study and severe disability levels were significant (just hypertension was marginally significant) and corroborate what has been reported in the geriatric literature about the role of chronic conditions and multimorbidity on the disablement process (6, 51). However, it must be noted that we only explored the singular effect of each chronic condition on disability, and it remains possible that different combinations of these diseases increase the likelihood of severe disability (51).

Last but not least, women in our study showed higher severity levels of disability than men. Studies have shown that older women are more likely than men to become and remain disabled (52, 53). The explanations for these differences have been explored following two main approaches. One is based on gender differences related to the presence of chronic diseases and other health conditions. Under this approach, older women have higher rates of diseases directly related to disability (arthritis, depression, osteoporosis, dementia) and are more prone to the presence of highly disabling geriatric syndromes (sarcopenia, frailty, falls, and related fractures) than older men (54). The second emphasizes the role of social determinants in explaining gender differences among older adults regarding disability. It is now widely accepted that social determinants (which include SES, occupation, income, among others) contribute to gender-related inequities (55). Evidence in HICs and LMICs shows that age-adjusted disability prevalence rates are higher for women when social factors are considered (26, 56–58). One of the main hypotheses to explain this effect is that the accumulated disadvantages in education and income, together with the traditional role of care, which are impediments to the social mobility of women at early ages, persists in old age, enhancing its adverse effects on health and wellness (59). Despite the above approaches, little has been explored about gender differences in severity levels of disability. Subsequent studies, mainly longitudinal, should generate evidence on this issue.

### Strengths and Limitations

Even though the severity levels of disability have been estimated in HICs, there are no cross-country comparisons using harmonized instruments in LMICs. In that vein, our study is the first attempt to fill this gap. Besides this, the WHO SAGE study employed standardized instruments that were used in all six countries that increased the external validity of our findings and ensured comparability of the results across the SAGE countries.

Nevertheless, some limitations must be considered in this study. First, recall and survivor bias can be limitations for epidemiological studies with older adult participants. It could be, for example, that older adults with worse disability conditions have died at younger ages. If this was the case, our prevalence of severe disability would be underestimated since the “healthiest” older people were interviewed. Second, most of the variables in the SAGE study, including the disability questions, are self-reported. This may have led to overestimation or underestimation of the true prevalence of severe disability among older adults. In spite of this, consistent and similar prevalence rates have been reported in HICs. Third, it is possible that the pooling of country data to some extent masks observed patterns of severe disability and its associated factors within individual countries. However, the inclusion of a country dummy variable in our analysis highlights some contrasts between countries that could inform to policy makers. For example, compared with the other countries, India has the highest prevalence of severe disability, which adds evidence about a recognized public health concern in that country (60).

### Policy and Research Implications

Disability in older adults is a complex process that goes beyond the physical limitations. According to the WHO conceptual framework reflected in the ICF, disability involves biological and disease conditions that are integrated into a social and environmental context. Also, disability is not a stable condition—individuals can progress to a more critical state related to its level of severity. In that sense, future aging research could focus in older adults with severe disability since these people usually live with worse socioeconomic and health conditions as corroborated by the results of this study. Additionally, the measurement and study of disability severity could be a critical element to understand the causes and consequences of aging, as well as to plan health programs and services. This is an even more urgent action in LMICs due to the rapid growth of their aging populations and the limited resources that they will face in the short and mid-term (61, 62).

A consequence of severe disability in older adults is that their independence in daily activities, mobility, and social participation is reduced, as the results of our study show. With the current projections of rapid aging process in LMICs, the number of severely disabled older adults living in the community will increase, and in turn, the demand for long-term care services will rise. This could represent a threat to the financial sustainability of health systems and social services (63). Therefore, there is a perennial need for evidence-based public health policy to design prevention programs, modify interventions, or develop enabling environments that help severely disabled older adults continue living in the community to preserve their autonomy, dignity, and social participation. Some HICs, like Japan, have driven a promissory public policy to tackle this issue. Japanese long-term care insurance (*Kaigo Hoken*) has set a radical change from the traditional family-based care toward the socialization of older adults' care. This program includes medical care and welfare services with the aim to reduce the care burden of caregivers and maintain the functional status of older adults (64). Given that evidence in LMICs has shown that the care of older adults with chronic diseases or disability falls closely on family members (65), Japanese experience could be useful to design national or local programs to attend to older adults with some degree of disability.

## Data Availability Statement

The datasets analyzed during the current study are available in the WHO repository, http://apps.who.int/healthinfo/systems/surveydata/index.php/catalog/sage/about.

## Ethics Statement

All procedures performed in this study were reviewed and approved by the following Ethics Committes: the WHO Ethical Review Committee; Ethics Committee, Shanghai Municipal Centre for Disease Control and Prevention, Shanghai, China; Ethical Committee, University of Ghana Medical School, Accra, Ghana; Institutional Review Board, International Institute of Population Sciences, Mumbai, India; Ethics Committee, National Institute of Public Health, Cuernavaca, Mexico; Ethics Committee, School of Preventive and Social Medicine, Russian Academy of Medical Sciences, Moscow, Russia; and the Research Ethics Committee, Human Sciences Research Council, Pretoria, South Africa. All procedures were performed according to the 1964 Helsinki declaration and its later amendments or comparable ethical standards. Informed consent was obtained from all individual participants included in the study.

## Author Contributions

AS-R, BM-E, and AR-A contributed to the conception and design of this research. AS-R conducted the data analyses, interpreted the data, and wrote the first and subsequent drafts of the paper. AS-R, BM-E, AR-A, and AS contributed to the interpretation of findings and substantially revised the manuscript for important intellectual content. All authors contributed to the article and approved the submitted version.

## Conflict of Interest

The authors declare that the research was conducted in the absence of any commercial or financial relationships that could be construed as a potential conflict of interest.
