# Supplementary material for: Severity Levels of Disability Among Older Adults in Low- and Middle-Income Countries: Results From the Study on Global Ageing and Adult Health (SAGE)
Source: Front Med (Lausanne). 2020 Oct 15;7:562963. doi: 10.3389/fmed.2020.562963 (PMC7594509; doi:10.3389/fmed.2020.562963)
Supplement: Supplementary file 1 [file Data_Sheet_1.docx]

Supplementary Material

**Supplementary Table 1. Fit and classification indices for latent class analysis**

|  | **Number of classes** | | | | | |
| --- | --- | --- | --- | --- | --- | --- |
| **Fit index** | **1** | **2** | **3** | **4** | **5** | **6** |
| Loglikelihood | -369812.92 | -306060.18 | -292598.62 | -286453.72 | -283388.64 | -281347.27 |
| Adjusted BIC | 272103.98 | 144866.59 | 118211.55 | 106189.84 | 100327.75 | 96513.10 |
| Bootstrapped LRT, p‐value |  | 127505.48, p<0.01 | 26923.12, p<0.01 | 12289.8, p<0.01 | 6130.16, p=0.053 | 4082.74, p=0.101 |
| Entropy |  | 0.92 | 0.89 | 0.86 | 0.84 | 0.84 |

**Supplementary Table 2. Response proportions for each item of WHODAS 2.0**

|  | **Response proportions** | | | | |
| --- | --- | --- | --- | --- | --- |
| **Items** | **1** | **2** | **3** | **4** | **5** |
| 1. Standing for long periods such as 30 minutes? | 0.47 | 0.26 | 0.14 | 0.10 | 0.03 |
| 2. Taking care of your household responsibilities? | 0.60 | 0.23 | 0.10 | 0.05 | 0.02 |
| 3. Learning a new task, for example, learning to get to a new place? | 0.42 | 0.30 | 0.17 | 0.09 | 0.02 |
| 4. How much of a problem did you have joining in community activities (for example, festivities, religious or other activities) in the same way as anyone else can? | 0.67 | 0.19 | 0.08 | 0.04 | 0.02 |
| 5. How much have you been emotionally affected by your health problems? | 0.48 | 0.29 | 0.15 | 0.07 | 0.01 |
| 6. Concentrating on doing something for ten minutes? | 0.65 | 0.21 | 0.09 | 0.04 | 0.01 |
| 7. Walking a long distance such as a kilometre? | 0.48 | 0.22 | 0.13 | 0.11 | 0.06 |
| 8. Washing your whole body? | 0.85 | 0.10 | 0.03 | 0.02 | 0.01 |
| 9. Getting dressed? | 0.87 | 0.08 | 0.03 | 0.01 | 0.01 |
| 10. Dealing with people you do not know? | 0.65 | 0.17 | 0.09 | 0.06 | 0.02 |
| 11. Maintaining a friendship? | 0.75 | 0.15 | 0.07 | 0.02 | 0.01 |
| 12. Your day‐to‐day work? | 0.70 | 0.17 | 0.08 | 0.04 | 0.01 |

Note. Response categories: 1=none; 2=mild; 3=moderate; 4=severe; 5=extreme or cannot do.

**Supplementary Figure 1. Distribution of WHODAS 2.0 score for four- and five-classes solution**

**Supplementary Figure 2. Probabilities of severe and extreme difficulty for activity in each of its six domains of WHODAS 2.0 by latent class**

**Supplementary Figure 3. Semantic interpretation for the 4 groups identified in the latent class analysis**
